# A RP-UFLC Assay for Protein Tyrosine Phosphatases: Focus on Protein Tyrosine Phosphatase Non-Receptor Type 2 (PTPN2)

**DOI:** 10.1038/srep10750

**Published:** 2015-06-04

**Authors:** Romain Duval, Linh-Chi Bui, Jérémy Berthelet, Julien Dairou, Cécile Mathieu, Fabien Guidez, Jean-Marie Dupret, Jan Cools, Christine Chomienne, Fernando Rodrigues-Lima

**Affiliations:** 1 Université Paris Diderot, Sorbonne Paris Cité, Unité de Biologie Fonctionnelle et Adaptative, CNRS UMR 8251, 75013, Paris, France; 2 Université Paris Diderot, Sorbonne Paris Cité, INSERM UMR_S1131, Institut Universitaire d’Hématologie, 75010 Paris, France; 3 VIB Center for the Biology of Disease, Leuven, Belgium; 4 Service de Biologie Cellulaire, Assistance Publique des Hôpitaux de Paris (AP-HP), Hôpital Saint Louis, 75010 Paris, France

## Abstract

Protein tyrosine phosphatases (PTPs) are involved in numerous signaling pathways and dysfunctions of certain of these enzymes have been linked to several human diseases including cancer and autoimmune diseases. PTPN2 is a PTP mainly expressed in hematopoietic cells and involved in growth factor and JAK/STAT signaling pathways. Loss of function analyses in patients with mutation/deletion of the *PTPN2* gene and knock-out mouse models indicate that PTPN2 acts as a tumor suppressor in T-cell malignancies and as a regulator of inflammation and immunity. The use of sensitive and quantitative assays is of prime importance to better characterize the biochemical properties of PTPN2 and its biological roles. We report a highly sensitive non-radioactive assay that allows the measurement of the activity of purified PTPN2 and of endogenous PTPN2 immunoprecipitated on agarose beads. The assay relies on separation and quantitation by reverse-phase ultra fast liquid chromatography (RP-UFLC) of a fluorescein-labeled phosphotyrosine peptide substrate derived from the sequence of STAT1. The applicability and reliability of this approach is supported by kinetic and mechanistic studies using PTP inhibitors. More broadly, our PTPN2 assay provides the basis for the design of flexible methods for the measurement of other PTPs.

Many key cellular signaling events are regulated by tyrosine phosphorylation which relies on the biochemically opposing actions of protein kinase and protein phosphatases^1^,^2^. Protein tyrosine phosphatases (PTPs) are critical regulators that participate in multiple signaling transduction events implicated in gene transcription, cell growth, differentiation, metabolism and immune response^3^. It is now well established that perturbation of certain PTPs is involved in various human disorders such as cancer and auto-immune diseases^2^,^4^. Several PTPs are thus emerging as drug targets for common human diseases, including cancer, diabetes, arthritis and infectious diseases^5^.

PTPN2 (protein tyrosine phosphatase non-receptor type 2, also known as TC-PTP) is a cytosolic tyrosine phosphatase highly expressed in hematopoietic cells and established as an important modulator of growth factor and cytokine-induced signaling pathways. Members of the JAK/STAT signaling pathways and different receptor protein tyrosine kinases such as EGFR and VEGFR have been described as substrates of PTPN2^2^,^5^,^6^. In addition, PTPN2 deficiency in mice results in severe defects of the hematopoietic tissue (affecting lymphoid, myeloid and erythroid lineages) and in systemic inflammation. These disorders are fatal and KO mice succumb rapidly after birth^7^,^8^. In humans, focal deletion or inactivation of PTPN2 by non-sense mutations in T-cell leukemia and T-cell non-Hodgkin’s lymphoma have been reported recently^9^,^10^,^11^. Functional analyses confirmed that PTPN2 act as a tumor suppressor^10^. Indeed, decreased expression/activity of PTPN2 was shown to provide a proliferation advantage to leukemic cells due, at least in part, to increased activation of the JAK/STAT pathway^9^,^11^. Moreover, it has also been shown that the loss of PTPN2 may contribute to resistance of chronic myeloid leukemia cells to imatinib through the modulation of PTPN2-dependent signals downstream the BCR-ABL fusion protein^12^. More recently, PTPN2 was found to attenuate T-cell lymphopenia-induced proliferation highlighting a major mechanism by which T-cells responses are tuned to prevent autoimmune and inflammatory disorders^13^.

PTPN2 activity is thus a novel biomarker of various human diseases and the establishment of simple, sensitive and quantitative activity assays is crucial to better understand PTPN2 and its biological roles. In particular, these assays should help to identify substrates or modulators of PTPN2 and evaluate the catalytic status of the enzyme in cells or tissues. The most commonly used phosphatase assay to measure PTPs, in particular PTPN2, involves simple chromogenic or fluorogenic phosphate esters or the use of ^32^P-labeled phosphotyrosyl proteins or peptides^14^,^15^,^16^. The measurement of inorganic phosphate released from a phosphopeptide substrate has also been used^15^. Each of these assays has drawbacks such as the frequent handling of radioactivity, lack of sensitivity and/or specificity. Assays based on peptides derived from known protein substrates appear as the most pertinent^15^,^16^. However, most of these methods rely on phosphotyrosine mimics which may impact their binding to active site. In addition, most of these assays are not suitable (sensitivity of the assay to background phosphate) or not sensitive enough to measure low levels of endogenous PTPs (in crude extracts or in immunoprecipitates).

In the present study, we provide a novel non-radioactive assay that allows the measurement of the activity of recombinant purified PTPN2 and cellular PTPN2 immunoprecipitated on agarose beads. The assay relies on the rapid separation and quantitation by reverse-phase ultra fast liquid chromatography (RP-UFLC) of a fluorescein-labeled phosphotyrosine peptide substrate derived from the sequence of STAT1, a known substrate of PTPN2. The sensitivity, biological relevance and applicability of the assay are demonstrated by kinetic analyses, inhibitors evaluation as well as the measurement of activity of endogenous PTPN2 present in cell lysates. Our assay is thus suitable for the screening and characterization of regulatory molecules of PTPN2 and for assaying the activity of endogenous PTPN2 present in cells or tissues. Finally, we show that our flexible approach can be used to assay other PTPs.

## Results and Discussion

### Quantitation by RP-UFLC of a fluorescent-peptide substrate of PTPN2 and its dephosphorylated product

To set up the assay, recombinant human PTPN2 was expressed in *E. coli* as a 6 X His-tagged protein and purified to homogeneity (Supplementary Figure 1). Purified PTPN2 was functional as demonstrated by the dephosphorylation of the non-proteinaceous and non-specific chromogenic substrate *p*-nitrophenyl phosphate (pNPP)(Supplementary Figure 2)^15^.

Compared to conventional chromogenic phosphate derivatives, assays based on the more realistic peptidic substrates are more specific and versatile^16^. Therefore, to assess the PTP activity of PTPN2, we synthesized a fluorescein-conjugated peptide substrate derived from the sequence of the human transcription factor Stat1 (FAM-KGTG**Y**_**701**_IKTE-NH_2_ abbreviated as FAM-pStat1) and containing a tyrosine residue known to be dephosphorylated by PTPN2^17^. A dephosphorylated form of FAM-pStat1 corresponding to the “product peptide” was also synthesized and is referred to as FAM-Stat1. Presence of the fluorescein moiety allows for the specific and sensitive detection and quantitation of both peptides by fluorescence. The assays were all carried out in 96-well plates and the samples were automatically processed for RP-UFLC using an autosampler.

**Figure 1:**
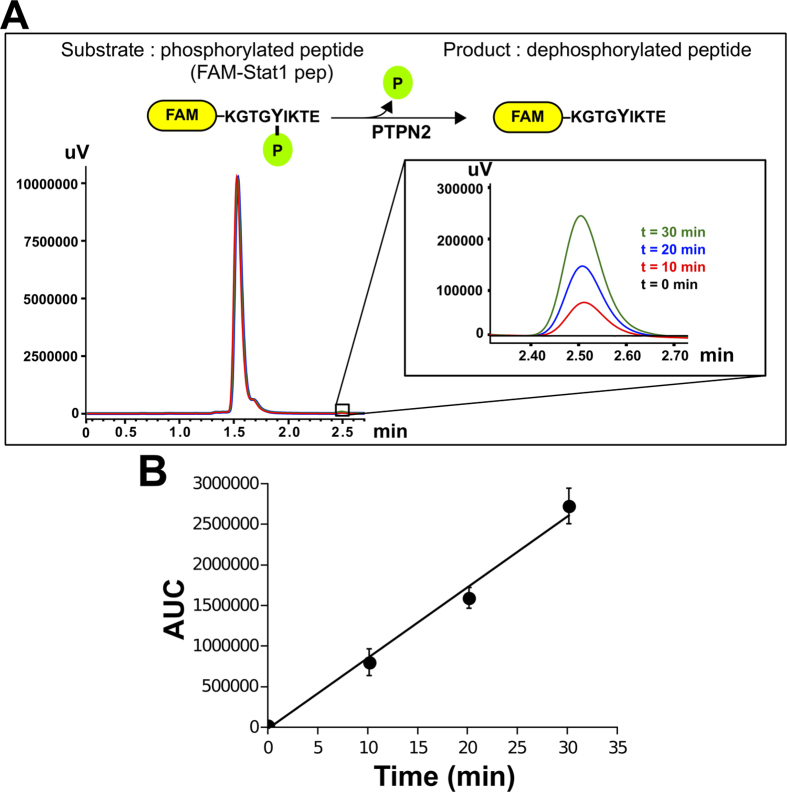
Initial velocity determination of PTPN2 reaction using FAM-pStat1 peptide and RP-UFLC. (**A**) Typical RP-UFLC chromatograms of the reaction mixture analyzed at different time points are shown. FAM-pStat1 peptide (substrate) and its dephosphorylated product (FAM-Stat1) are shown. The elution of both peptides was monitored by using the FAM-specific fluorescence emission at 530 nm (excitation: 485 nm). (**B**) Initial velocity determination. The AUC (area under the curve) of the FAM-Stat1 peptide (product) peak was plotted *versus* time of the reaction, and the slope was taken as initial velocity (V_i_).

To show that purified PTPN2 displayed tyrosine-phosphatase activity towards FAM-pStat1 peptide, the enzyme was incubated with the peptide substrate for different times and the mixture analyzed by reverse phase UFLC using a C18 column. The chromatograms clearly showed that FAM-pStat1 (retention time of 1.6 min) and its dephosphorylated product FAM-Stat1 (retention time of 2.5 min) were readily separated and detected by RP-UFLC (Figure 1). The time required for the RP-UFLC separation is shorter (2.7 min) than conventional HPLC methods that take at least 5 min per run^18^,^19^. More importantly, the amount of dephosphorylated product (FAM-Stat1) increased linearly with time as confirmed by integration (Area Under the Curve, AUC) of the FAM-Stat1 peak (Figure 1A inset and Figure 1B). For optimal initial rate (V_i_) measurements, the assays were conducted with an excess of FAM-pStat1 over enzyme concentration to ensure that less than 5% of the substrate is enzymatically converted into product during the assay^20^. The initial rate of the reaction (Vi) is directly obtained from the slope of the plot of the AUC *versus* time of reaction (Figure 1B). A calibration curve established with various known concentrations of FAM-Stat1 peptide was used to transform AUC units into concentrations of product.

### Kinetic analysis of PTPN2 and inhibition studies with vanadate, hydrogen peroxyde and phenyl vinyl sulfonate

**Figure 2:**
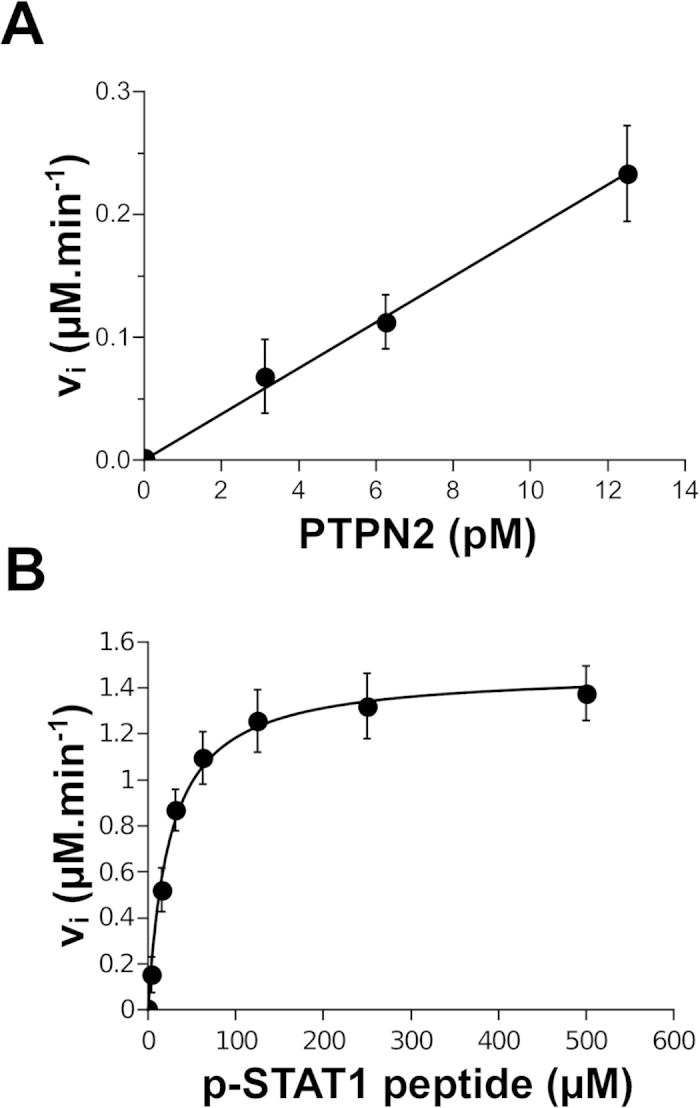
Kinetic analysis of recombinant PTPN2 using FAM-pStat1 peptide and RP-UFLC. (**A**) Ackermann-Potter plot. Several concentrations of purified PTPN2 were incubated with 50 μM of FAM-pStat1 peptide. Initial velocities (V_i_) were determined and plotted against PTPN2 concentrations. (**B**) Michaelis-Menten saturation curve for FAM-pStat1 peptide dephosphorylation by PTPN2. Various concentration of FAM-pStat1 peptide (0-500 μM) were incubated with purified PTPN2 (50 pM). Initial velocities (V_i_) were determined and fitted to Michaelis-Menten equation.

Further kinetic analyses were carried out in presence of different concentrations of purified PTPN2 (Figure 2A) or with different concentrations of FAM-pStat1 (Figure 2B). As shown in Figure 2A, the initial rates determined by the assay were proportional to enzyme concentrations. Although the incubations were carried out for only 30 min at 37 °C, the assay proved to be highly sensitive since low pM concentrations of the enzyme could be readily measured. Concentrations lower than 1 pM of PTPN2 could be detected with higher incubation times (data not shown). In general, non radioactive PTP assays detect enzyme activity mainly in the nM range^14^,^15^,^16^,^19^. By using fluorescence detection of peptidic substrates, this RP-UFLC assay achieves high sensitivity and overcomes the disadvantages of the radioactive approaches^19^,^21^. Accordingly, the detection limit for the FAM-pStat1 and FAM-Stat1 peptide was low and estimated to be equal to 10 fmol. In addition, as our assay is insensitive to background phosphate, it can be conducted in phosphate buffer which has been reported to be the optimal choice for PTP activity measurements^16^.

As shown in Figure 2B, incubation of PTPN2 with increasing concentrations of FAM-pStat1 led to increasing initial rates following Michaelis-Menten saturation kinetics. Non-linear regression fitting of the data to Michaelis-Menten equation revealed a K_m_ for FAM-pStat1 of 25 μM which is close to K_m_ values reported for phosphorylated peptidic substrates of PTP1B, a prototypic member of the PTP family which is the closest homolog of PTPN2 (these sister PTPs display 71% amino acid identity in their catalytic domain and form the NT1 subtype in the phylogenetic classification system of PTPs)^1^,^6^. The *k*_cat_ value of PTPN2 for FAM-pStat1 was equal to 490 s^−1^. Reported values for kinetic parameters of PTPs (and in particular for PTPN2) remain scarce and not easy to compare between the different PTPs. In addition, Michaelis-Menten parameters such as K_m_ may vary with the experimental conditions used in the different assays. However, our data conform with typically observed K_m_ and k_cat_ values for PTPs with peptidic substrates (K_m_ values ranging from 10 to hundreds μM and k_cat_ values ranging from 1 to hundreds s^–1^^16^,^22^.

All members of the PTP family share the same catalytic mechanism which depends on an essential cysteine residue at the active site^23^. Oxidation and/or modification of this catalytic cysteine leads to enzyme inhibition. In addition, it is now recognized that oxidative inhibition of PTPs such as PTPN2 promotes tyrosine phosphorylation and thus enhances signaling responses^4^,^24^. Inhibition studies were carried out with three well-known inhibitors of PTPs, *i.e*. vanadate (Na_3_VO_4_), hydrogen peroxyde (H_2_O_2_) and phenyl vinyl sulfonate (PVSN). Vanadate is a phosphate analog and is generally considered to bind as a transition state analog to phosphoryl transfer enzymes such as phosphatases^25^. H_2_O_2_ is known to be a key intracellular second messenger in many signaling pathways. PTPs (including PTNP2) are now recognized as important targets of this reactive oxygen specie which inhibits PTPs through oxidation of their catalytic cysteine residue^1^,^24^,^26^. Aryl vinyl sulfonates such as PVSN are specific inhibitors of PTPs that react covalently with the catalytic cysteine thereby impairing enzyme activity. These chemicals are used in applications aimed at characterizing functionaly PTPs^27^.

**Figure 3:**
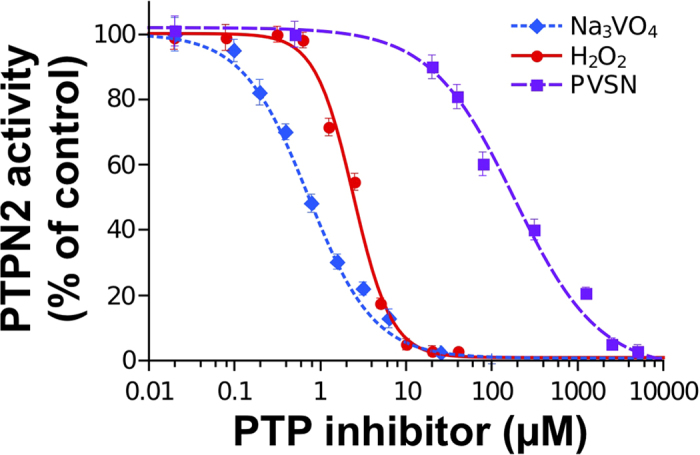
Analysis of known PTP inhibitors on purified PTPN2 using FAM-pStat1 and RP-UFLC. Various concentrations of orthovanadate (Na3VO4), hydrogen peroxyde (H_2_O_2_) or phenyl vinyl sulfonate (PVSN) were preincubated with purified PTPN2 (50 pM final) for 10 min at 37 °C. FAM-pStat1 peptide (50 μM final) was added to start the reaction and the residual PTPN2 activity was determined using RP-UFLC. Assays were carried out in triplicate. IC_50_ values (inhibitor concentration required to reduce enzyme activity by 50%) were determined by non linear regression.

**Figure 4:**
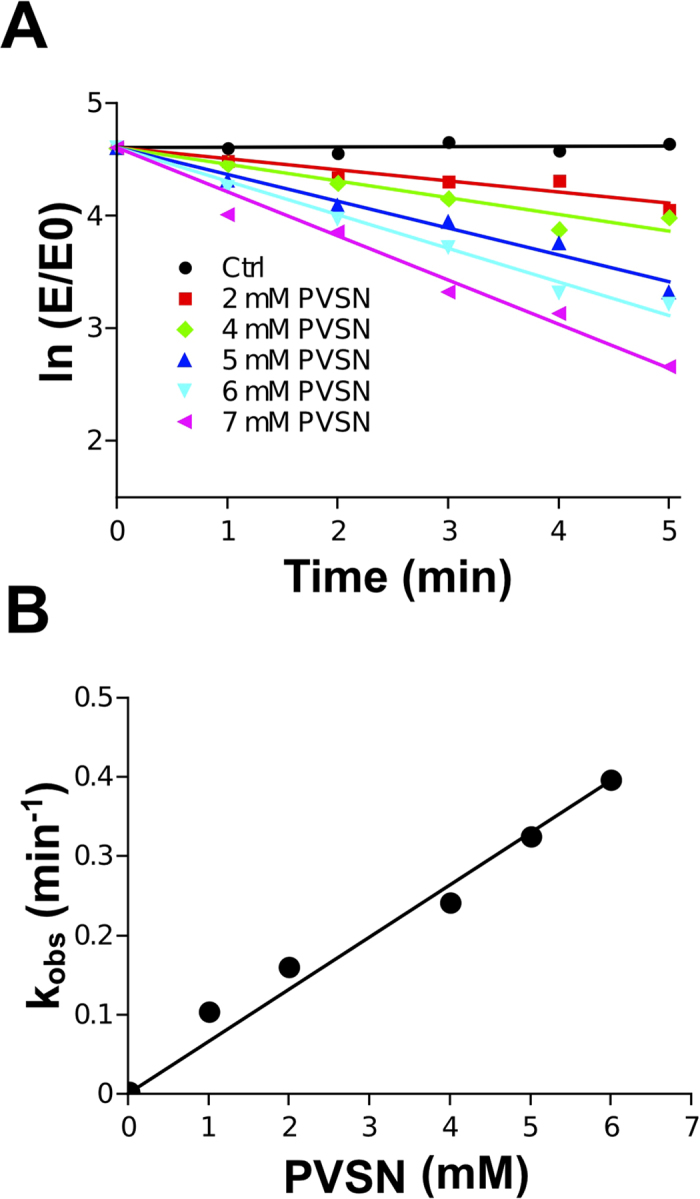
Kinetic analysis of PVSN-dependent inhibition of PVSN using FAM-pStat1 and RP-UFLC. (**A**): Plots of the natural logarithm of the percentage residual PTPN2 activity *versus* time for each PVSN concentration. The apparent first-order inhibition constants (*k*_obs_) for the different concentration of PVSN were determined from the slopes of linear regression lines. (**B**): *k*_i_ determination. The *k*_obs_ were plotted against PVSN concentrations and the second-order inhibition constant (*k*_i_) was determined from the slope of the linear regression lines.

As expected, purified PTPN2 was found to be inhibited in a dose-dependent manner by the three inhibitors (Figure 3). Strong inhibition of the enzyme (>90% inhibition) was achieved with low micromolar concentrations of vanadate and H_2_O_2_ (IC_50_ values close to 1 and 3 μM). Higher concentrations of PVSN (above 1 mM) were needed to obtain 90% inhibition of the enzyme (IC_50_ = 180 μM). These results are in agreement with data obtained with other PTPs (including human PTP1B) and carried out with different assays approaches (peptide-based or chromogenic/fluorogenic phosphate esters-based assays)^24^,^25^,^26^,^27^. Further inhibition analyses were carried out with PVSN to confirm that our assay is suitable for the precise characterization of enzyme inhibition mechanisms. As PVSN is reported to be a mechanism-based irreversible inhibitor of PTPs, we examined the effect of this inhibitor on PTPN2 activity as a function of time and concentration using our RP-UFLC approach^27^. We found that PVSN inhibited PTPN2 in a time- and concentration-dependent first-order process which is in accordance with the properties of PVSN (Figure 4A). Similar results were reported with other PTPs including PTP1B^27^. Further analysis of the pseudo-first order rate constant of inhibition (k_obs_) as a function of inhibitor concentration showed that the data fitted well to a line passing at the origin thus indicating that the inhibition of PTPN2 by PVSN occured through a single bimolecular process (Figure 4B). The second-order rate constant for the inhibition (k_i_) of PTPN2 by PVSN was found to be 60 M^−1^. min^−1^. This value is close to the k_i_ found for the inhibition of YopH PTP by PVSN (168 M^−1^. min^−1^)^27^.

The robustness and reproducibility of our RP-UFLC/peptide-based PTPN2 assay was further evaluated by calculating the statistical Z´-factor^28^. We obtained a Z´ value of 0.66 which corresponds to an assay of good quality, characterized by a significant gap between positive and negative controls and a low deviation between replicates^28^.

Altogether our kinetic studies indicate that our assay is reliable for kinetic and mechanistic analyses of PTPN2 and for the identification and/or characterization of inhibitors.

### Measurement of endogenous PTPN2 activity in cells exposed or not to H_2_O_2_

**Figure 5:**
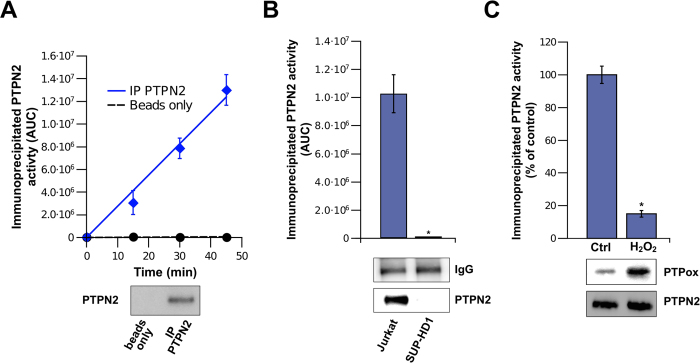
Measurement of cellular PTPN2 activity in immunoprecipitates using FAM-pStat1 and RP-UFLC. (**A**) Determination of the activity of immunoprecipitated endogenous PTPN2. Endogenous PTPN2 was immunoprecipitated from Jurkat cells extracts using an polyclonal anti-PTPN2 antibody (IP PTPN2). The immunobeads were incubated with FAM-pStat1 (50 μM final) for different time prior to RP-UFLC analysis. Beads not coated with anti-PTPN2 antibody were used as control (beads). The AUC of the dephosphorylated-FAM-pStat1 peptide peak was plotted *versus* time. Proteins bound to immunobeads (IP PTPN2) or control beads were also analyzed by Western blot using a monoclonal anti-PTPN2 antibody. (**B**) Immunoprecipitation studies with Jurkat cells and PTPN2-deficient human cells (SUP-HD1). Immunoprecipitations were carried out in parallel with extracts from Jurkat and SUP-HD1 cells using the polyclonal anti-PTPN2 antibody. The immunobeads were incubated with FAM-pStat1 (50 μM final) for RP-UFLC analysis. Proteins bound to immunobeads were analyzed by Western blot using the monoclonal anti-PTPN2 antibody. (**C**) Inhibition of endogenous PTPN2 by H_2_O_2_. Jurkat cells were left untreated (control) or treated 30 min with 20 μM H_2_O_2_. Endogenous PTPN2 was immunoprecipitated with a polyclonal anti-PTPN2 antibody and the immunobeads were incubated with FAM-pStat1 peptide (50 μM final) and the residual PTPN2 activity was determined (normalized to the control). *p < 0.05. Immunobeads-bound proteins from treated (H_2_O_2_) and not treated (control) were analyzed by Western blot using a monoclonal anti-PTPN2 antibody (PTPN2) or a monoclonal anti-oxydized PTP (after stripping of the membrane). *p < 0.05 *versus* non-treated cells (Mann-Whitney U test, Prisme software).

Sensitive and quantitative assessment of endogenous PTPN2 activity in different cellular contextes is critical to better understand the regulation and the role of this important human PTP. Most of the commonly used PTP assays are not suitable (or have not been evaluated) to quantify specific PTP activities in biological samples. We took advantage of the high sensitivity of our assay and of the commercial availibility of specific antibodies against PTPN2 to measure the activity of the endogenous enzyme present in cell extracts. As several PTPs, such as PTP1B and PTPN2 have overlapping substrate specificities, immunoprecipitation of a specific PTP from complex biological samples is of prime importance to assay specifically the activity of a given PTP^1^,^15^. As shown in Figure 5A, significant activity towards p-Stat1 could be readily and rapidly measured using PTPN2 enzyme immunoprecipitated from lysates of Jurkat cells (1.10^6^ cells). PTPN2 activity was also readily determined in bone marrow and spleen from C57BL/6J mice (data not shown). Similar immunopurification studies were carried out with SUP-HD1 cells which are known to be devoid of PTPN2^10^. Contrary to the data obtained with Jurkat cells, no PTPN2 protein nor PTP activity towards FAM-pStat1 could be found in the immunobeads (Figure 5B) thus supporting the biological relevance of the assay. Further experiments were carried out with Jurkat cells that were exposed to H_2_O_2_. This reactive oxygen species is known to oxidize the catalytic of cysteine of cellular PTPs (including PTPN2) leading to an inactive enzyme^23^,^24^. As shown in Figure 5B, PTPN2 immunoprecipitated from H_2_O_2_-treated cells displayed low activity when compared to PTPN2 immunoprecipitated from control cells (15% residual activity). In parallel, Western-blotting analyses using specific antibodies against oxidized-PTPs confirmed that loss of endogenous PTPN2 activity was due to oxidation of its catalytic cysteine (Figure 5B, lower panel). These experiments support the fact that our assay can be used to determine the activity of endogenous PTPN2 in different biological settings. To circumvent the existence of substrate overlap between PTPs and to measure the activity of a specific endogenous PTP, the use of an immunoprecipitation step prior to enzyme assay is needed^15^. Although this approach relies on the availability of specific antibodies that do not impair the activity of the PTP enzyme, it still remains so far the best way to measure the activity of a particular PTP present in a biological sample without extensive biochemical purification or the identification/design of a specific substrate^15^. Alternatively, omission of the immunoprecipitation step allows to assess the overall PTP activity towards p-Stat1 present in a biological lysate. In addition, the present PTPN2 assay can be used to do substrate profiling (by using other peptide sequences as putative substrates) and to assay other PTPs (either purified or immunoprecipitated).

In conclusion, the assay described here offers a new alternative to the methods currently used to measure the activities of purified PTPs, in particular PTPN2. This flexible assay is simple, highly sensitive, quantitative and uses more realistic substrates. In addition, the 96-well plate format is well suited for the screening and the kinetic and mechanistic characterization of substrates or inhibitors.

## Methods

### Materials

All reagents were purchased from Euromedex (France) or Sigma-Aldrich (France) unless otherwise stated. UFLC-grade solvents were from Sigma-Aldrich or Biosolve (France). A 9 amino acid peptide derived from the sequence of human Stat1 protein (a known physiological substrate of PTPN2^17^) was synthesized, conjugated to fluorescein amidite (FAM) on its *N*-terminus and modified by amidation (NH_2_) on its C-terminus (Proteogenix, France). A tyrosine residue known to be dephosphorylated by PTPN2 was incorporated into the peptide as a phosphotyrosine. The peptide (which is referred to as FAM-pSTAT1) was as follows: FAM-KGTGY_701_IKTE-NH_2_ (where the bold tyrosine is a phosphotyrosine). A dephosphorylated form of FAM-pSTAT1 were the phosphotyrosine is replace by a tyrosine was also used and referred to as FAM-Stat1.

### Expression and purification of recombinant human PTPN2 enzyme

The complementary DNA (cDNA) coding for the full length human PTPN2 (TC45 variant) was cloned into pET28a plasmid and used to produce 6xHis-tagged PTPN2 protein in *E. coli*. The pET28a-PTPN2 plasmid was transformed into *Escherichia coli* 21 (DE3) for production and purification of the PTPN2 enzyme. Briefly, transformed bacterial cells were grown overnight at 16 °C in the presence of 0.5 mM of IPTG. Cells were harvested by centrifugation and resuspended in lysis buffer (20 mM Tris HCl pH 8, 300 mM NaCl, 0.2% Triton X-100, 1 mg/ml lysozyme and protease inhibitors). The lysate was subjected to sonication on ice and pelleted (12,000g; 30min). The supernatant was incubated with His-select nickel resin (Sigma) in the presence of 20 mM imidazole for 2h at 4 °C. Resin was poured into an empty column and washed with washing buffer (20 mM Tris-HCl pH 7.5, 150 mM NaCl and 35 mM imidazole). PTPN2 was eluted in elution buffer (20 mM Tris-HCl pH 7.5, 150 mM NaCl and 300 mM imidazole). After reduction with 10 mM DTT (15 min on ice), purified PTPN2 was applied to a PD-10 column (GE Healthcare) equilibrated with 20 mM Tris-HCl, pH 7.5, 150 mM NaCl. Protein concentration and purity were assessed by Bradford reagent (Bio-rad) and by SDS-PAGE. Proteins were kept at –80 °C until use.

### RP-UFLC-based separation and quantification of the fluorescein-labeled peptide substrate of PTPN2 (FAM-pStat1) and its dephosphorylated product

The purity and identity of FAM-pStat1 and its dephosphorylated form (FAM-Stat1) peptide was initialy assessed by reverse-phase UFLC (Prominence Shimadzu UFLC [ultra fast liquid chromatography] system interfaced with LabSolutions software). Samples were injected onto a Shim-pack XR ODS column (length = 100 mm, internal diameter = 2 mm, particle size = 2.2 μm) (Shimadzu) at 40 °C. The mobile phase used for the separation consisted of two eluents: solvent A was water with 0.12% trifluoacetic acid (TFA), and solvent B was acetonitrile with 0.12% TFA. Compounds were separated by an isocractic flow (80% A/20% B) rate of 0.6 ml/min. FAM-pStat1 and its dephosphorylated form (product) were monitored by fluorescence emission (λ = 530 nm) after excitation at λ = 485 nm and quantified by intregration of the peak absorbance area, employing a calibration curve established with various know concentrations of peptides.

### Kinetic analysis and inhibition of purified PTPN2 by orthovanadate (Na_3_VO_4_), hydrogen peroxyde (H_2_O_2_) and phenyl vinyl sulfonate (PVSN)

Assay were performed in a 96-wells ELISA plate in a total volume of 100 μL consisting of phosphatase buffer (100 mM sodium acetate pH 6, 1 mM DTT), FAM-pStat1 peptide (up to 500 μM final) and purified PTPN2 (up to 50 nM final). Briefly, samples containing the enzyme were preincubated with phosphatase buffer and the reaction was started with the addition of FAM-pStat1. At different time points (up to 30 min), 100 μL of HClO_4_ (15% in water) was added to stop the reaction, and 20 μl were automatically injected into the RP-UFLC column.

For inhibition studies, PTPN2 (50 pM final) was preincubated with the different inhibitors (at various concentrations for 30 min at 37 °C). The reaction was started by the addition of FAM-pStat1 (50 μM final). The mixture (total volume 100 μl) was incubated for 10 min at 37 °C prior to RP-UFLC analysis.

To determine the second-order rate inhibition constant of PVSN, PTPN2 was preincubated with various concentration of PVSN under pseudo first-order conditions. Briefly, PTPN2 (500 pM final) was incubated with PVSN (1-5 mM final) in 100 mM sodium acetate pH 6. At various time intervals, aliquots were removed and assayed for residual activity after dilution (10 times with phosphatase buffer). The equation rate of inhibition of recombinant PTPN2 by PVSN can be represented as :

ln [E/E_0_] = *k*_obs_ x t.

Provided that PVSN is present in substantial excess, the apparent first order inhibition rate constants (*k*_obs_ = *k*_inact_ x [PVSN]) can be calculated for each PVSN concentration from the slope of the natural log (ln) of percent residual activity plotted against time. The second-order rate constant (*k*_inact_) was determined from the slope of *k*_obs_ plotted against PVSN concentrations. Kinetic data were plotted and fitted using Qtiplot software (http://www.qtiplot.com/).

### Cell culture, whole cell extract and exposure to hydrogen peroxyde

Jurkat cells (immortalized human T lymphocyte cells) were maintained in RPMI 1640 medium supplemented with 10% heat-inactivated fetal bovine serum and 1 mM L-glutamine. SUP-HD1 cells (immortalized human Hodgkin lymphoma cells) were maintained in McCoy’s medium supplemented with 20% heat-inactivated fetal bovine serum. The culture medium was renewed every three days bringing the cell concentration to ~2 × 10^5^ cells/ml. To make whole-cell extracts, cells were first washed 3 times with PBS and lysed in PBS pH 7.5, 0.2% Triton X100 and protease inhibitors for 15 min in ice. After centrifugation (12000 x g, 15 min), the supernatant was taken for further analysis. Protein concentration in extracts were measured using the Bradford assay.

For the endogenous PTPN2 inhibition experiments, Jurkat cells were incubated 30 min at 37 °C either with PBS (control) or in PBS containing 20 μM H_2_O_2_ (final). Cell extracts were made as described above.

### PTPN2 immunoprecipitation and activity assay

For PTPN2 immunoprecipitation, 200 μl of whole-cell extracts (5 mg/ml) were incubated overnight with 2 μg of polyclonal rabbit anti-PTPN2 antibody (Sigma-Aldrich, reference : SAB4200249) at 4 °C. Samples were then rocked for two hours at 4° C in presence of 1 mg of protein A–Agarose (Sigma-Aldrich). The immunobeads were harvested by centrifugation, washed 3 times with PBS 0.1% Triton X100 and incubated with 400 μL of phosphatase buffer containing 50 μM FAM-pStat1 peptide at 37 °C. Every 15 min, beads were spun down by centrifugation (2000 x g, 5 min), 50 μl of supernatant were taken and mixed with 50 μL of HClO_4_ (15% in water) prior to RP-UFLC analysis.

At the end of the activity assay, beads were harvested and boiled in 40 μL of Laemmli Sample Buffer (without reducing agents). Eluates were further used for Western blot analysis with an anti-PTPN2 mouse monoclonal antibody (R&D system) or an anti-oxydized PTP monoclonal mouse antibody (R&D system).

## Additional Information

**How to cite this article**: Duval, R. *et al*. A RP-UFLC Assay for Protein Tyrosine Phosphatases: Focus on Protein Tyrosine Phosphatase Non-Receptor Type 2 (PTPN2). *Sci. Rep*. **5**, 10750; doi: 10.1038/srep10750 (2015).

## Supplementary Material

Supplementary Information
