# Supplementary material for: A RP-UFLC Assay for Protein Tyrosine Phosphatases: Focus on Protein Tyrosine Phosphatase Non-Receptor Type 2 (PTPN2)
Source: Sci Rep. 2015 Jun 4;5:10750. doi: 10.1038/srep10750 (PMC4455150; doi:10.1038/srep10750)
Supplement: Supplementary Information [file srep10750-s1.pdf]

Supplementary information

A RP-UFLC assay for protein tyrosine phosphatases: focus on protein tyrosine phosphatase non-receptor type 2 (PTPN2)

Romain Duval[a], Linh-Chi Bui[a], Jérémy Berthelet[a], Julien Dairou[a], Cécile Mathieu[a], Fabien Guidez[b], Jean-Marie Dupret[a], Jan Cools[c], Christine Chomienne[b,d] and Fernando Rodrigues-Lima[a, e]

[a] Université Paris Diderot, Sorbonne Paris Cité, Unité de Biologie Fonctionnelle et Adaptative, CNRS UMR 8251, 75013, Paris, France

[b] Université Paris Diderot, Sorbonne Paris Cité, INSERM UMR_S1131, Institut Universitaire d'Hématologie, 75010 Paris, France

[c] VIB Center for the Biology of the Disease, Leuven, Belgium

[d] Service de Biologie Cellulaire, Assistance Publique des Hôpitaux de Paris (AP-HP), Hôpital Saint Louis, 75010 Paris, France.

[e] Correspondence and request for materials should be addressed to FRL (fernando.rodrigues-lima@univ-paris-diderot.fr)

Supplementary Figure 1. Expression and purification of recombinant PTPN2

(A) SDS–PAGE analysis of recombinant PTPN2. 5 and 10 µg (lanes 1 and 2, respectively) of purified recombinant PTPN2 were subjected to SDS–PAGE under reducing conditions and stained with Coomassie Blue R-250.

(B and C) Western blot analysis of recombinant PTPN2. 100 and 200 ng (lanes 1 and 2, respectively) of purified proteins were subjected to SDS–PAGE under reducing conditions and Western blot analysis using a monoclonal antibody raised against PTPN2 (B) or 6×His tag (C).

Supplementary Figure 2. PTPN2 activity assay using pNPP substrate

Recombinant PTPN2 enzyme was incubated at different concentrations in 100 mM sodium acetate (pH 6) in the presence of 10 mM (final) pNPP. The absorbance at 405 nm was continuously measured every 15 s.

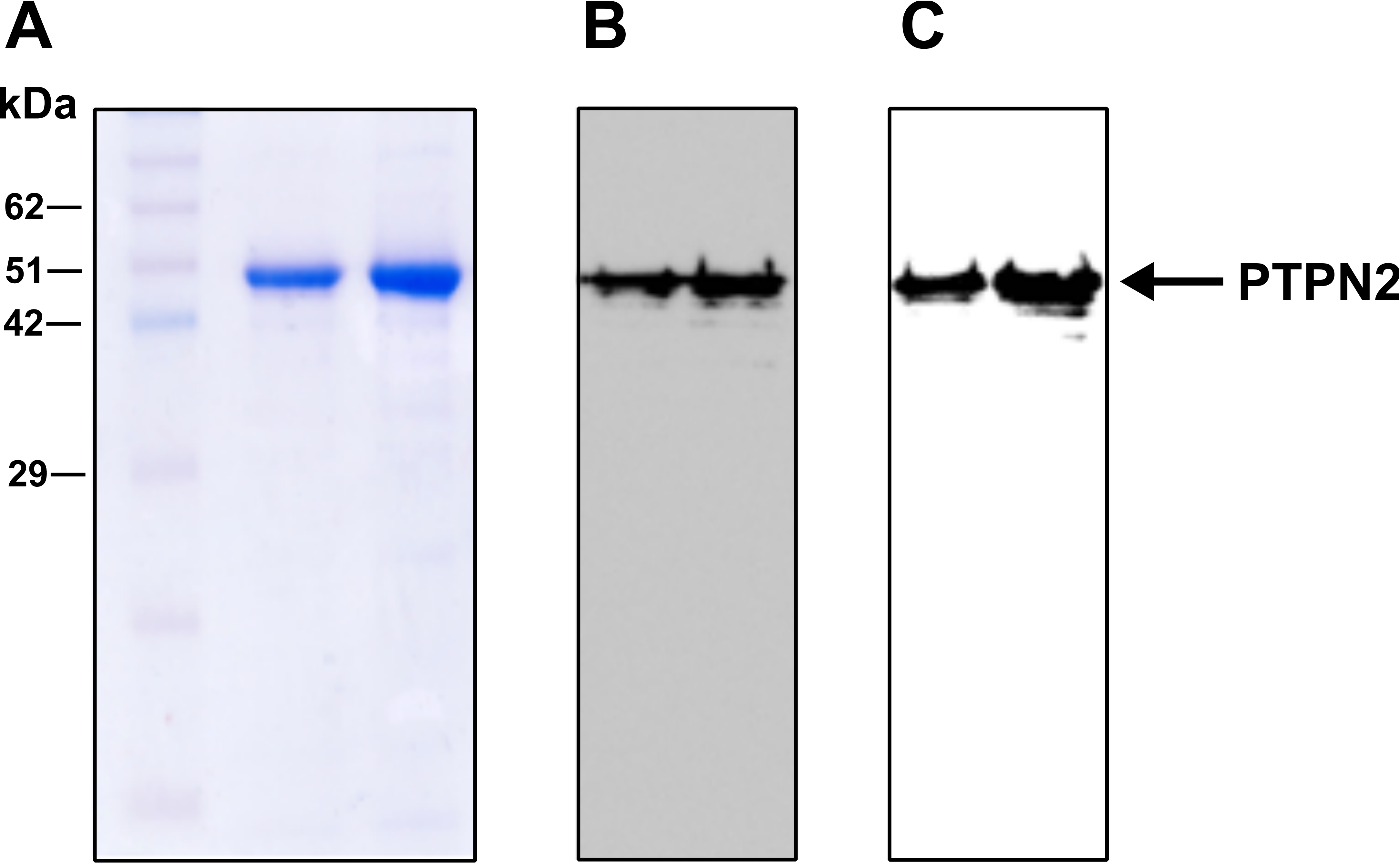

Supplementary figure 1

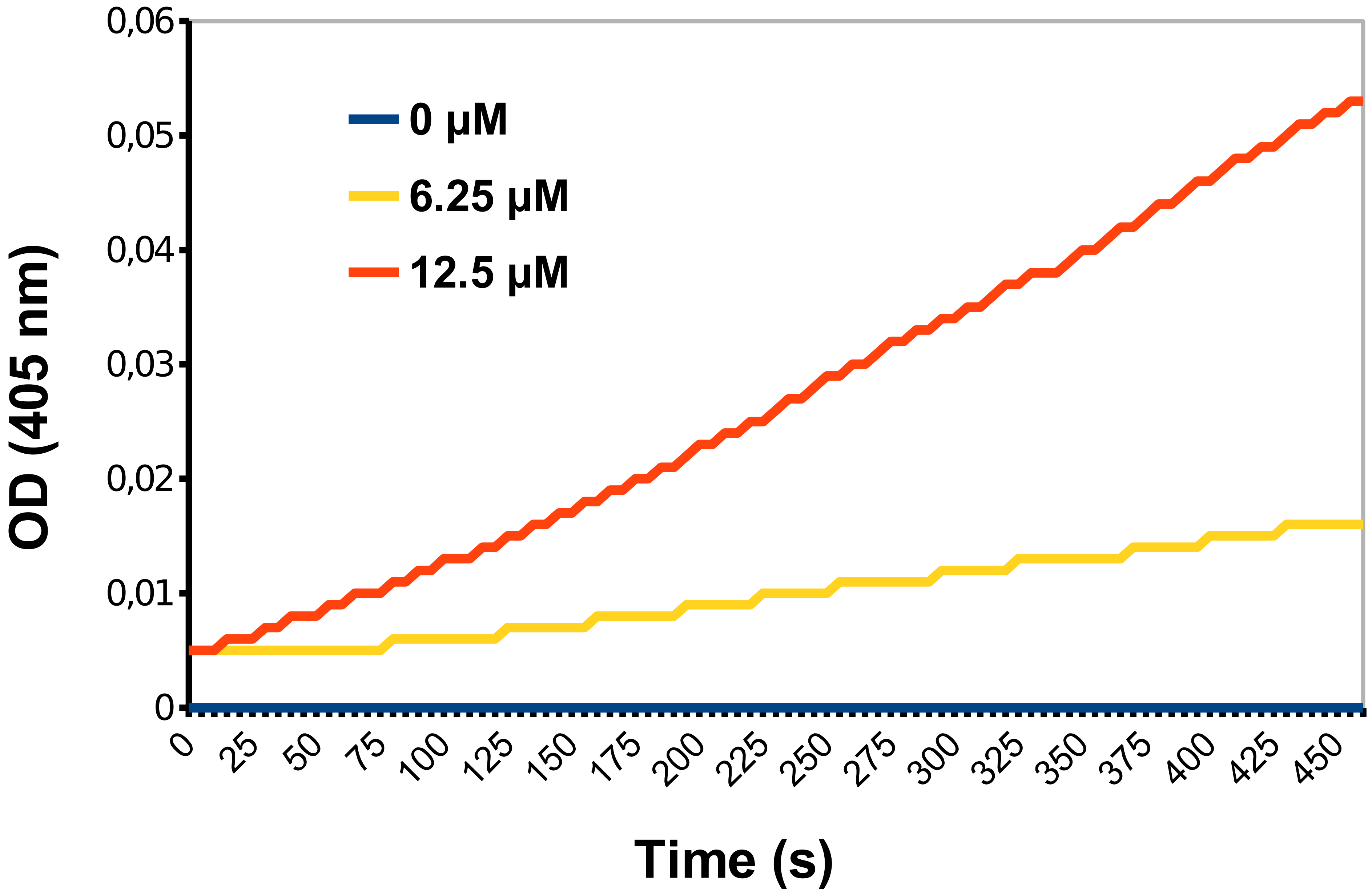

Supplementary figure 2
